# Psychotic disorders in migrant population

**DOI:** 10.1192/j.eurpsy.2021.1940

**Published:** 2021-08-13

**Authors:** P. Felgueiras, P. Barbosa

**Affiliations:** Psychiatry, Vila Nova de Gaia Hospital Center, Vila Nova de Gaia, Portugal

**Keywords:** socio-developmental-cognitive model, acculturation, psychosis, migrants

## Abstract

**Introduction:**

One of the defining features of the modern world is a large scale migration that occurs due to a range of factors, from political conflicts to personal and voluntary reasons. This process can cause a severe disruption in individuals’ biography and can be followed by a large period of adaptation and a phenomenon of acculturation. Surprisingly, there is little research on the impact of migration and settlement on risk of psychosis.

**Objectives:**

Regarding a clinical case, we aim to enphasize the current evidence about the risk of psychotic disorders in migrants.

**Methods:**

We present a qualitative review of this topic using the Pubmed database.

**Results:**

27 years old portuguese female, with hyperthymic temperament and history of depressive episode. Her process of migration in 2016 was motivated by an academic purpose. In context of stressful life events she developed psychotic symptoms - messianic and persecutory delusions, with visual and auditory hallucinations.

**Conclusions:**

There is an increased risk of psychosis among migrant population that is well documented. This is even the main risk factor with the exception of a family history of psychosis. The risk can be explained by socio-demographic and psychological features, factors involving the migration process, and socio-occupational environment in the host country. A socio-developmental-cognitive model theorize how exposure to a stressful environment and social defeat interacts with underlying genetic risk to create an enduring liability to psychosis. These findings can help in important decisions about mental health resources and accessibility, including protocols to identify and treat migrants at higher risk of mental diseases.

**Disclosure:**

No significant relationships.

